# Utilising an accelerated Delphi process to develop consensus on the requirement and components of a pre-procedural core robotic surgery curriculum

**DOI:** 10.1007/s11701-022-01518-2

**Published:** 2023-02-09

**Authors:** Joshua Richard Burke, Christina A. Fleming, Martin King, Charlotte El-Sayed, William S. Bolton, Chris Munsch, Deena Harji, Simon P. Bach, Justin W. Collins

**Affiliations:** 1 The Association of Surgeons in Training, Royal College of Surgeons of England, London, England, UK; 2 Robotics and Digital Surgery Initiative, Royal College of Surgeons of England, London, England; 3 Leeds Institute Medical Research, University of Leeds, Leeds, UK; 4 The Royal College of Surgeons, Dublin, Ireland; 5 Craigavon Area Hospital, Craigavon, Northern Ireland; 6 Technology Enhanced Learning Directorate of Innovation, Digital and Transformation, Health Education England, London, England; 7 Academic Department of Surgery, University of Birmingham, Birmingham, UK; 8 Department of Colorectal Surgery, Manchester University NHS Foundation Trust, Manchester, UK; 9 University College London, Division of Surgery and Interventional Science, Research Department of Targeted Intervention, London, UK; 10 Wellcome/ESPRC Centre for Interventional and Surgical Sciences (WEISS), UK, University College London, London, UK

**Keywords:** Robotic surgery, Robot-assisted surgery, Training, Curriculum, Delphi method

## Abstract

Robot-assisted surgery (RAS) continues to grow globally. Despite this, in the UK and Ireland, it is estimated that over 70% of surgical trainees across all specialities have no access to robot-assisted surgical training (RAST). This study aimed to provide educational stakeholders guidance on a pre-procedural core robotic surgery curriculum (PPCRC) from the perspective of the end user; the surgical trainee. The study was conducted in four Phases: P1: a steering group was formed to review current literature and summarise the evidence, P2: Pan-Specialty Trainee Panel Virtual Classroom Discussion, P3: Accelerated Delphi Process and P4: Formulation of Recommendations. Forty-three surgeons in training representing all surgical specialties and training levels contributed to the three round Delphi process. Additions to the second- and third-round surveys were formulated based on the answers and comments from previous rounds. Consensus opinion was defined as ≥ 80% agreement. There was 100% response from all three rounds. The resulting formulated guidance showed good internal consistency, with a Cronbach alpha of > 0.8. There was 97.7% agreement that a standardised PPCRC would be advantageous to training and that, independent of speciality, there should be a common approach (95.5% agreement). Consensus was reached in multiple areas: 1. Experience and Exposure, 2. Access and context, 3. Curriculum Components, 4 Target Groups and Delivery, 5. Objective Metrics, Benchmarking and Assessment. Using the Delphi methodology, we achieved multispecialty consensus among trainees to develop and reach content validation for the requirements and components of a PPCRC. This guidance will benefit from further validation following implementation.

**Supplementary Information:**

The online version contains supplementary material available at 10.1007/s11701-022-01518-2.

## Introduction

The adoption of robotic-assisted surgery (RAS) across multiple surgical specialities continues to expand globally. Between 2018 and 2019, there was a 20% increase in hospital acquisition of the Da Vinci robot and with growth sustained throughout the COVID-19 pandemic [1–3]. With the development of robotic technology, the surgical robotics market is expected to grow to a value of £18 billion by 2025 [4]. This increased adoption of robotic technology has coincided with the emergence of several new robotic surgery systems entering clinical practice including CMR Surgical’s ‘Versius’ robot and Medtronic’s ‘Hugo’ robot. In the United Kingdom (UK) between 2000 and 2019 Lam et al. [5] demonstrated that around 25% of NHS trusts had a robotic platform and in the same time frame the number of cases performed robotically increased by 400%. Following this study there has been a landmark £20 m investment by NHS Scotland [6] and a further £18 m by NHS Wales into robotics platforms [7].

Despite this expansion and increasing adoption of RAS in clinical practice recognising the perceived benefits of improved dexterity, ergonomics and improved anatomical access [8], surgical trainee exposure to robotic-assisted surgical training (RAST) is limited. It is estimated that > 70% of current UK and Irish surgical trainees have had no access to any form of robotic surgery during their surgical training to date [9, 10]. This same cohort of trainees, however, do place significant value on RAST prior to completion of training as they feel it will be important for their future consultant practice and they would support its incorporation into the UK and Irish surgical training curriculae [11–13]. As with any new technology, appropriate training to safeguard patients and optimise outcomes is crucial but with the increasing adoption of robotic surgery, there is a clear requirement for safe and standardised RAST during surgical training.

**Fig. 1:**
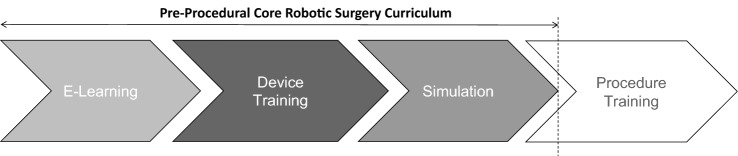
There was 95.3% agreement that following successful completion of core robotic surgery training, a trainee should be able to proceed to procedural training in their preferred specialty

Training in robotic surgery can generally be divided into four sequential stages: e-learning, device training, simulation and finally procedure training (Fig. 1). In current surgical practice, RAST is significantly industry led and focused on procedure-based training for consultant and senior stage surgical trainees and fellows and design of RAST has been replete of trainee stakeholder input [11].

Furthermore, basic skills training requires access to hardware and has historically been industry driven, while procedural focused training has been curtailed by the lack of expertise from UK and Ireland surgical trainers or replete of trainee surgeon consultation [9, 10, 13, 14]. However, with increasing adoption of robotic surgery and requirement for robotic proficient surgeons in the future there is a requirement to increase surgical trainee literacy, knowledge and skills in robotic surgery through structured and validated training at all stages of training, so that trainees are appropriately skilled and prepared for procedure-based training when that opportunity arises [15]. This form of a pre-procedural core robotic surgery training to proficiency level in basic robotic skills training can be pan-specialty relevant and appropriate for integration in parallel to current surgical training structures [16]. Current Specialty Training curricula in the UK and Ireland do not reference the role or requirement for RAST in surgical training [17]. The aim of this Delphi process was to explore surgical trainee consensus on the requirement for and components of, a pre-procedural core robotic surgery curriculum (PPCRC) for use in current surgical training in the UK and Ireland.

## Methodology

This Delphi consensus consisted of four phases, where each phase informed the subsequent phase. The Delphi method is a well-established approach to answering a research question through the identification of a consensus view across subject experts—in this instance surgical trainees in elected speciality positions. It allows for reflection among participants, who are able to reconsider their opinion based on the anonymised opinions of their peers [18].

Available evidence was reviewed which informed pan-specialty trainee panel discussion to develop the Delphi consensus questionnaire. Finally, an accelerated Delphi process was delivered to formulate these guidance and recommendations. Descriptive statistics describing demographics were performed using Stata/SE16 (Stata Corp, UK).

### Phase 1: Evidence acquisition

A steering group was formed consisting of trainee representatives, a robotic surgeon and robotic surgery trainer to review and summarise the current evidence base for RAST curriculae across all surgical specialities through review of peer-reviewed publications, association guidelines and industry published literature.

**Fig. 2:**
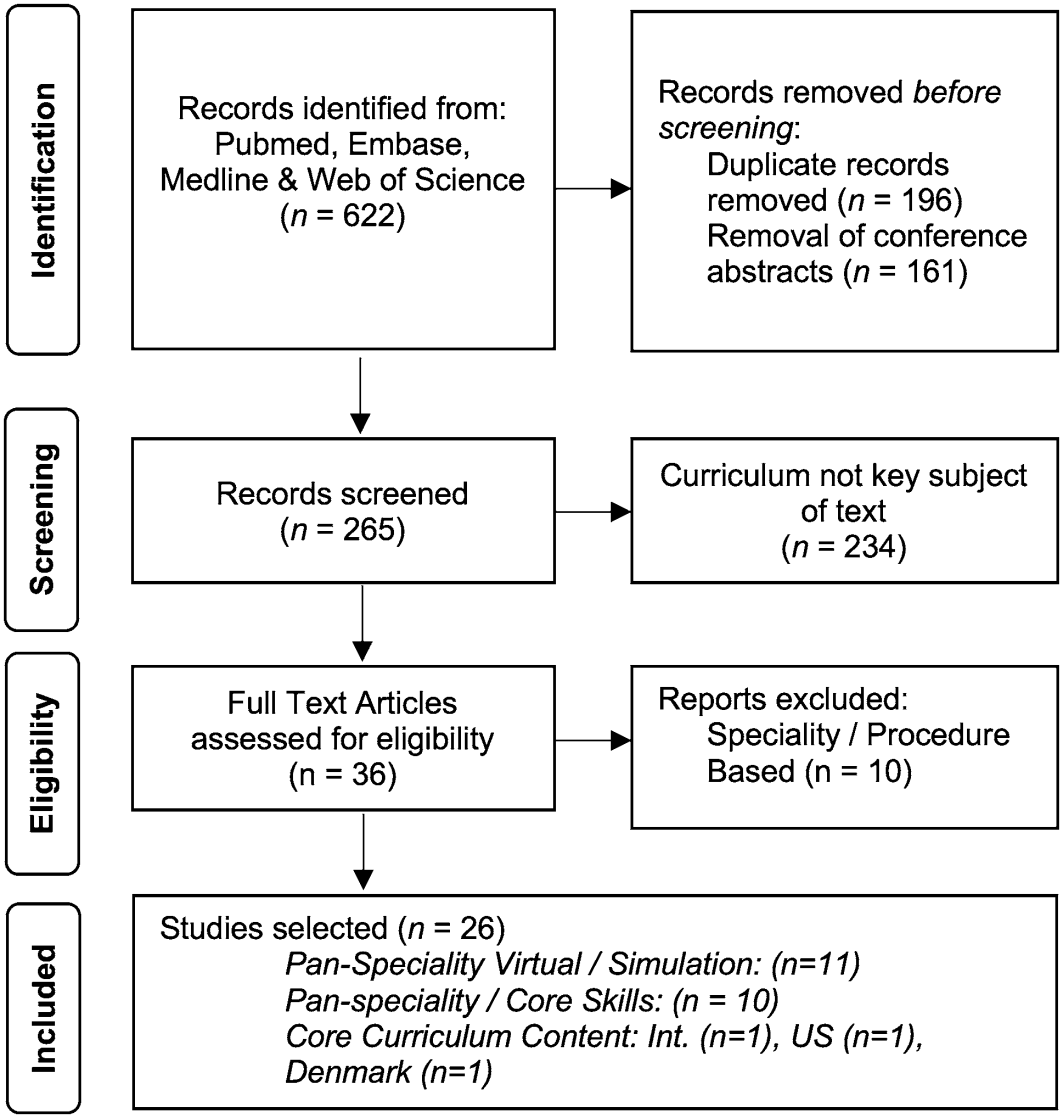
PRISMA flow diagram summarising the study selection process. *PRISMA* Preferred Reporting Items for Systematic Reviews and Meta-analyses

A systematic review of the current literature and was completed independently by three individuals (JB, CF and JC). The systematic review was not registered with PROSPERO as it does not meet the registration criteria but is reported in accordance with the Preferred Reporting Items for Systematic Reviews and Meta-analyses (PRISMA) statement [19]. The authors reviewed current published literature on PubMed, EMBASE, and Web of Science databases for full-text English-language articles published between 1995 and 2020, using the keywords “Robotic” OR “Robot-assisted”, AND “Surgery” OR “Surgical” AND “Curriculum” OR “Programme”. Additional significant studies cited in the reference list of selected papers were evaluated. The reviewers independently selected papers for detailed review after evaluating the abstract and, if necessary, the full-text manuscript. Potential discrepancies were resolved by open discussion. The electronic search yielded a total of 622 potential articles. Figure 2 summarises the selection process.

Multiple prospective studies were identified, which made recommendations as to the contents of a generic robotics curriculum [20–44]. Overall, the quality of available studies was moderate to low reported as per the GRADE quality assessment framework [45]. Available evidence consists largely of expert opinion, consensus statements, and small qualitative studies. One opinion piece and was identified that focused specifically on a PPCRC in the UK and Ireland [43] and one international consensus piece with limited trainee involvement [46]. Twenty-six articles were selected by the core team and placed into five categories to inform discussion on Access and Context (*n* = 6), Experience and Exposure to Robotic Surgery (*n* = 8), Curriculum Components (*n* = 22), Objective Metrics (*n* = 13), Benchmarking and Assessment (*n* = 21). Eighteen papers were considered in more than one category.

### Phase 2: Pan-specialty trainee panel virtual classroom discussion

43 Trainees in elected trainee representative roles covering all surgical specialties from the council of The Association of Surgeon of Training (ASiT) were invited and agreed to participate. This participating group represented all surgical specialties and all levels of surgical training from medical student to post-Certification of Completion Training (CCT) fellow. ASiT is the largest trainee organisation in the UK and Ireland representing over 4500 members from all 15 trainee speciality associations, grades and training regions and is a registered charity (1,196,477). This panel of surgical trainee stakeholders convened and discussed the themes identified in Phase 1 over two 1.5-h structured discussion sessions.

This was conducted through audio and visual recording of presentation, mini-poll and open discussion using the UCL Virtual Software Platform (University College London, London) [47]. Given the study was run during the COVID-19 pandemic participants were asked to rate the impact of the pandemic on their current training using a Likert scale of 0 (No Disruption) to 5 (Significant Disruption) as an indication of general disruption to current surgical training which could influence opinion on surgical training as a whole.

### Phase 3: Internet survey and accelerated Delphi process

Following Phase 2, an accelerated Delphi methodology was used to quantify consensus in the participating group. This was performed electronically using Google Forms (Google, USA). The Delphi was generated fallowing a qualitative content-analysis [48] (performed by JB and CF) of Phase 2 recordings and distributed to all Phase 2 panel members. Delphi content was divided into 7 Sections as follows: (1) Demographics, (2) Access and Context (3) Experience and Exposure to Robotic Surgery, (4) Relevance to Individual, (5) Curriculum Components, (6) Target Groups and Delivery, (7) Objective Metrics, Benchmarking and Assessment (Supplementary Material 1). An accelerated three round electronic consensus exercise, over three consecutive days (planned a priori), using the Delphi methodology was then conducted [49]. Participants were asked to indicate their ‘agreement/disagreement’ with the proposed themes and criteria generated during Phase 2. Survey items with ≥ 80% consensus were removed from the subsequent round of the survey with the consensus threshold achieved disseminated to all participants. Repeated iterations of anonymous voting continued over three rounds, where an individual’s vote in the next round was informed by knowledge of the panel’s results in the previous round. For inclusion in the final recommendations, each survey item had to have reached group consensus (≥ 80% agreement) by the end of the three survey rounds (Supplementary Material 2—> 1). Items that did not achieve consensus were also discussed in Phase 4.

### Phase 4: Generation of recommendations

The study steering group summarised and reported the recommendations within this manuscript based on the consensus results of the Delphi process. The authorship of this paper is comprised of ASiT, The Royal College of Surgeons of England Robotics and Digital Surgery (RADAR) committee and Health Education England and Industry representatives who support the recommendations. Those Phase 3 items which did not reach consensus and are not included in the recommendations but were felt to be important by the steering group have been taken forward in further qualitative work by Health Education England and RCS England.

## Results

A 100% (43/43) response rate was received from participants in all three rounds. Following conclusion of the three rounds of the Delphi process 97 of 151 items achieved consensus (≥ 80% agreement or disagreement). There was 97.7% agreement that a standardised PPCRC would be advantageous to training and that independent of speciality, there should be a common approach (95.5% agreement). Tables 1, 2, 3, 4, 5 summarise the five themes identified when considering a PPCRC and detail those statement which reached 80–100% agreement using the Delphi process and the levels of agreement reached. A comprehensive list of all statements can be found in supplementary tables.

**Table 1:** Statements achieving consensus in the ‘Experience and Exposure to Robotic Surgery’ theme (*n* = 7)

Item	Statement	Agree (%)	Disagree (%)	Achieved consensus	Round achieved
*Experience and Exposure to Robotic Surgery*					
1	Robotic surgery can support an increase in delivery of minimally invasive surgery	**95.5**	4.5	Yes	1
2	Robotic surgery can enable microsurgical techniques in surgery	**97.7**	2.3	Yes	1
3	Robotic surgery can enable automated data collection on individual surgical performance	**93.0**	7.0	Yes	1
5	Robotic surgery will be relevant to your preferred speciality	**95.3**	4.7	Yes	1
6	You foresee robotic surgery as part of your future consultant practice	**81.4**	18.6	Yes	2
	*Integration of robotic surgery training could have a negative impact on your overall surgical training due to*				
7.1	Consultants still on robotic learning curve resulting in less learning opportunities	**86.0**	14.0	Yes	2
7.5	Compete with more valuable learning opportunities in current training environment	18.6	**81.4**	Yes	1

Numbers in bold represent > 80% consensus

**Table 2:** Statement achieving consensus in the ‘Access and Context’ theme (*n* = 12)

Item	Statement	Agree (%)	Disagree (%)	Achieved consensus	Round achieved
*Access and context*					
8	Where robotic surgery training is not available in all hospitals, a deanery/regional-based structure should be established for delivering training in robotic surgery to increase access	**95.3**	4.7	Yes	1
9	Where robotic surgery training is available in hospitals, a deanery/regional-based structure should be established for delivering training in robotic surgery and further increase access	**83.7**	16.3	Yes	1
10	Monthly access to a regional hub for core robotic surgery training is reasonable	**86.0**	14.0	Yes	2
11	Regional hubs should be supervised by robotic surgery trainers if they were an option	**93.0**	7.0	Yes	1
12	Individual robotic surgery trainers should be accredited	**93.0**	7.0	Yes	1
13	Training centres and training hospitals should be assessed and accredited via a recognised education entity, like a royal college or society	**90.7**	9.3	Yes	1
	Deanery/regional base approach to robotic surgery training should comprise				
14.1	*Device training in regional hub*	**86.0**	14.0	Yes	1
	During which times should trainees attend regional hubs if they were available through				
15.1	*Time-tables as part of working day*	**88.4**	11.6	Yes	2
15.2	*Approved educational leave*	**81.4**	18.6	Yes	3
15.3	*Zero days*	4.7	**95.3**	Yes	1
15.4	*Free time*	4.7	**95.3**	Yes	1
	A reasonable distance to travel to a regional hub is				
17.1	< 20 km	27.9	72.1	–	–
17.2	< 50 km	**83.7**	16.3	Yes	3
17.3	< 100 km	2.3	–	–	–

Numbers in bold represent > 80% consensus

**Table 3:** Statement achieving consensus in the ‘Curriculum Components’ theme (*n* = 41)

Item	Statement	Agree (%)	Disagree (%)	Achieved Consensus	Round achieved
*Curriculum components*					
	Components of a core robotic surgery curriculum should include				
18.1	*Virtual simulation training and e-learning*	**90.7**	9.3	Yes	1
18.2	*Baseline evaluation*	**83.7**	16.3	Yes	2
18.3	*Device training*	**88.4**	11.6	Yes	1
18.4	*Dry lab training*	**86.0**	14.0	Yes	1
18.8	*History of robotics*	18.6	**81.4**	Yes	1
18.9	*Live operating*	16.3	**83.7**	Yes	1
19	Division of robotic surgery training into different progressive phases to include: e-learning, device training, basic skills and procedural training is an effective approach	**95.3**	4.7	Yes	1
20	A core robotic surgery curriculum should include: e-learning, device training and basic skills	**97.7**	2.3	Yes	1
21	Following successful completion of core robotic surgery training, a trainee should be able to proceed to procedural training in their preferred specialty	**95.3**	4.7	Yes	1
22	All training should include objective metrics to assess progression to a defined level	**97.7**	2.3	Yes	1
23	Core robotic surgery training should be linked to proficiency-based progression, with 'bench marking' of pass/fail levels?	**86.0**	14.0	Yes	2
24	Trainees benefit from validated objective scoring systems to provide consistent feedback	**93.0**	7.0	Yes	1
25	Benchmarking of an acceptable standard of performance for trainees should be defined within the curriculum	**88.4**	11.6	Yes	1
26	Trainees should pass core robotic training before commencing advanced procedural training	**95.3**	4.7	Yes	1
27	All training should include baseline evaluation for assessment of training needs	**93.0**	7.0	Yes	
28	Baseline evaluation could enable different entrance levels to the core training, that takes into account the individuals current exposure to robotic surgery training	**86.0**	14.0	Yes	1
29	For novice trainees, online e-learning and completion of a baseline evaluation should be a pre-requisite proceeding to 'practical' core robotic training in the form of device training and basic skills	**81.4**	18.6	Yes	1
	Baseline evaluation should include				
30.1	*VR Simulation modules*	**83.7**	16.3	Yes	1
	E-learning should include				
31.1	*Description of hardware in various systems*	**90.7**	9.3	Yes	1
31.2	*Info on patient selection and preparation*	**86.0**	14.0	Yes	1
31.3	*Info on trouble shooting*	**90.7**	9.3	Yes	1
31.4	*How to dock*	**93.0**	7.0	Yes	1
31.8	*History and development of robotic surgery*	**18.6**	81.4	Yes	1
	Technical core robotic skills training should include				
32.1	*Two-handed movements*	**97.7**	2.3	Yes	1
32.2	*Camera direction*	**97.7**	2.3	Yes	1
32.3	*Basic movements*	**95.3**	4.7	Yes	1
32.4	*Tissue dissection*	**81.4**	18.6	Yes	1
32.5	*Knot tying*	**83.7**	16.3	Yes	1
33	Formal device training should be mandatory in a core robotic surgery curriculum	**90.7**	9.3	Yes	1
34	Training on each type of robotic surgery device is required before operating on a patient	**93.0**	7.0	Yes	1
35	Non-technical skills training should be a component of a core robotic surgery curriculum	**88.4**	11.6	Yes	1
	Non-technical skills should include				
36.1	*Situation awareness training*	**90.7**	9.3	Yes	1
36.2	*Operative team training*	**86.0**	14.0	Yes	2
36.3	*Communication*	**88.4**	11.6	Yes	2
36.4	*Emergency scenarios*	**83.7**	16.3	Yes	1
37	Operative team training should be a component of a core robotic surgery curriculum	**90.7**	9.3	Yes	1
	Operative team training should include				
38.1	*Docking*	**92.9**	7.1	Yes	1
38.2	*Emergency scenarios*	**90.5**	9.5	Yes	1
38.3	*Bedside assistance*	**85.7**	14.3	Yes	1
39	Non-technical skills training and team training should be evaluated with a scoring system?	**83.7**	16.3	Yes	3
40	Non-technical skills and team training can be sufficiently assessed with NOTSS	**88.4**	11.6	Yes	1

Numbers in bold represent > 80% consensus

**Table 4:** Statement achieving consensus in the ‘Target Groups and Delivery’ theme (*n* = 22)

Item	Statement	Agree (%)	Disagree (%)	Achieved consensus	Round achieved
*Target groups and delivery*					
41	A standardised core robotic training curriculum will be advantageous to training	**97.7**	2.3	Yes	1
42	Independent of specialty, there should be a common approach for core robotic surgery training	**95.3**	4.7	Yes	1
43	A core robotic surgery training programme should bring you to point of procedural training	**88.4**	11.6	Yes	1
	A core robotic curriculum should be available to the following				
44.1	*Core surgical trainees (or equivalent)*	**88.4**	11.6	Yes	1
44.2	*Registrars (or equivalent)*	**97.7**	2.3	Yes	1
44.3	*Fellows (or equivalent)*	**83.7**	16.3	Yes	1
44.4	*Robot naïve surgeons*	**83.7**	16.3	Yes	1
44.5	*Laparoscopic surgeons*	**81.4**	18.6	Yes	1
44.9	*Foundation year doctors*	16.3	**83.7**	Yes	1
	A core robotic curriculum should consider the experience of the following				
45.1	*Core surgical trainees (or equivalent)*	**90.7**	9.3	Yes	1
45.2	*Registrars (or equivalent)*	**95.3**	4.7	Yes	1
45.3	*Fellows (or equivalent)*	**81.4**	18.6	Yes	1
45.4	*Robot naïve surgeons*	**91.4**	8.6	Yes	1
45.5	*Laparoscopic surgeons*	**86.0**	14.0	Yes	1
45.6	*Open Surgeons*	**83.7**	16.3	Yes	1
45.8	*Extended surgical team*	18.6	**81.4**	Yes	1
45.9	*Foundation year doctors*	14.0	**86.0**	Yes	1
45.10	*Medical student*	7.0	**93.0**	Yes	1
46	Does robotic surgery only have a role in senior years of training? (Answer—NO)	**90.7**	9.3	Yes	2
47	Robotic surgery training should only be delivered post-CCT (Answer—DISAGREE)	**93.0**	7.0	Yes	1
48	Training in robotic surgery is beneficial in earlier years of surgical training (Answer—AGREE)	**90.7**	9.3	Yes	1
	Training in robotic surgery is beneficial in early years of training due to				
50.1	*Familiarity with basic principles in robotics*	**83.7**	16.3	Yes	1

Numbers in bold represent > 80% consensus

**Table 5:** Statement achieving consensus in the ‘Objective Metrics, Benchmarking and Assessment’ theme (*n* = 15)

Item	Statement	Agree (%)	Disagree (%)	Achieved consensus	Round achieved
*Objective metrics, benchmarking and assessment*					
51	Cases performed robotically should be accepted for indicative numbers for index cases in surgical training	**83.7**	16.3	Yes	1
52	Should standard logbook programmes facilitate an option for recording robotic approach to standard surgical procedures	**88.1**	11.9	Yes	1
53	Surgical curricula should reference the role of robotic surgery and guide on its place in training	**90.7**	9.3	Yes	1
54	Current curricula approved Procedure-Based Assessments (PBAs) should be adjusted (where appropriate) to be suitable to assess robotic approach to index cases	**90.7**	9.3	Yes	1
55	Specialty-relevant index procedure PBAs should be completed in simulation prior to live-operating?	**81.4**	18.6	Yes	2
56	Completion of core robotic surgery skills training should be considered as an approved surgical training course for certification	**83.7**	16.3	Yes	1
57	Trainees should receive a 'sign-off' following completion of core robotic surgery skills	**95.3**	4.7	Yes	1
	Final assessment for ‘sign-off’ should include				
58.1	*VR simulation modules*	**97.7**	2.3	Yes	2
59	*Core robotic surgery skills training should be formally built into surgical curriculum?*	**86.0**	14.0	Yes	2
60	*Robotic surgery training could have any negative impact on your overall surgical training*	**83.7**	16.3	Yes	3
	Technical core robotic skills training assessments should include				
61.1	*ISCP OBAs*	**81.0**	19.0	Yes	1
61.5	*GEARS*	7.0	**93.0**	Yes	1
62	*Videos should be analysed with a validated standardised objective scoring system*	**90.7**	9.3	Yes	1
	Scoring systems for video analysis should include				
63.5	*ABS operative performance rating*	2.3	**97.7**	Yes	1
63.6	*At least two 'experts' should analyse and review video performance*	**81.4**	18.6	Yes	1

Numbers in bold represent > 80% consensus

### Participant demographics

**Fig. 3:**
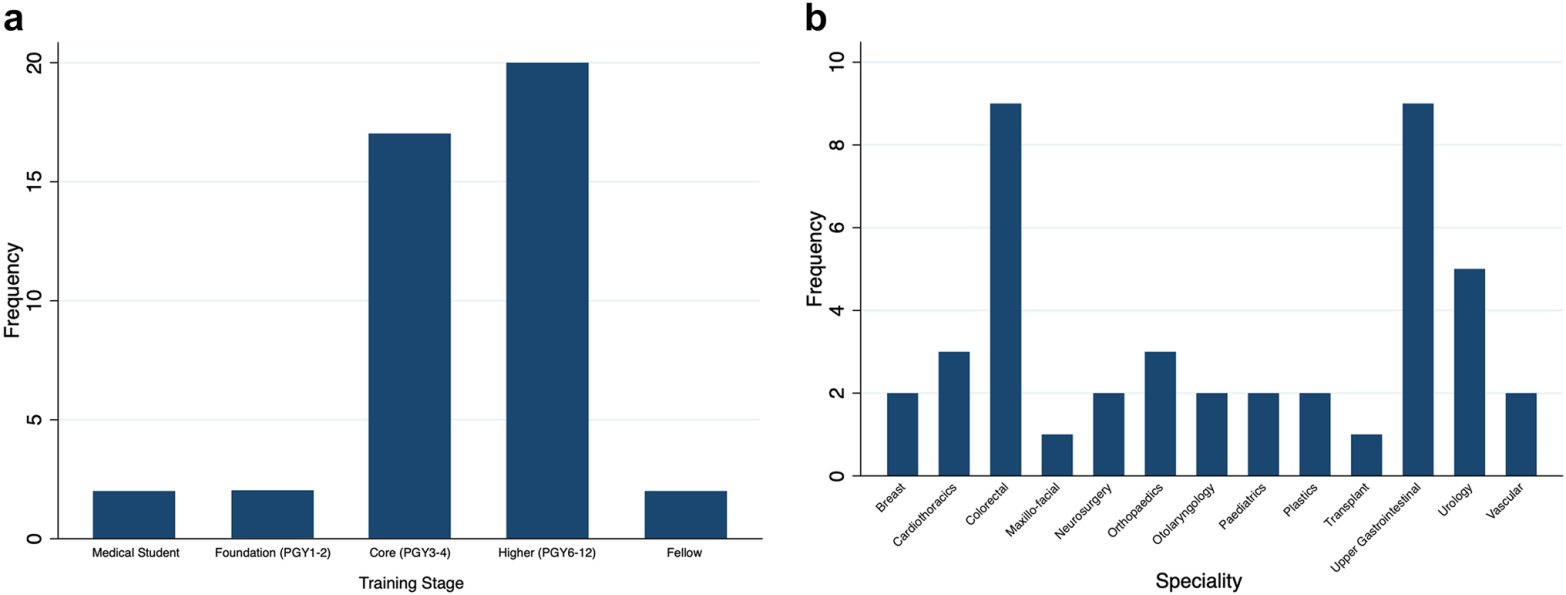
Training stage and preferred surgical speciality (*n* = 43)

Forty-three elected surgical trainee representatives participated representing all surgical subspecialties (Fig. 1). 25 (58%) were female and 18 (42%) were male. The majority of participants were higher surgical trainees (39.5%) with all training grades represented (Fig. 3). Three participants (7%) were less than full-time trainees and six (14%) were Academic Trainees. All training regions (Deaneries) were represented with a maximum of five (11.6%) trainees from any given deanery. The mean Likert score of current impact of COVID-19 on training was 3.3 (SD ± 1.5) representing a subjective trainee reported snapshot opinion of the impact of the COVID-19 pandemic on training. 97% of respondents reported that the COVID-19 pandemic had a significant disruption to their training (75% reported severe disruption).

### Experience and exposure to robotic surgery

There was strong consensus that robotic surgery can support an increase in the delivery of minimally invasive surgery, enable microsurgical techniques, automated data collection and is relevant to future consultant practice across the majority of specialities (Table 1). It was perceived that robotic surgery may have a negative impact on overall surgical training due to current consultant colleague learning curves and through competing with more valuable learning opportunities in the current UK and Ireland training environment. There was no consensus on consultant learning curves effecting training or on the possible negative effect of less complex cases selected for robotic procedures that would have offered ideal open or laparoscopic training opportunities (46.5%).

### Access and context

There was clear support for a regional-based training infrastructure, where robotic surgery is not available in all hospitals within said region with access facilitated through timetabling as part of the working day or on approved educational leave. They should not be limited to zero/rest days and trainees personal time Both trainers and training centres should be accredited (Table 2).

### Curriculum components

Components of a PPCRC should include a baseline evaluation of skill, e-learning, with device training and basic skills training delivered in various simulation settings, including VR simulation and dry lab training. Division of the curriculum into these phases was deemed an effective approach (Table 3). Following successful completion of core robotics surgery training a trainee should be able to progress onto procedural training in their preferred speciality (Fig. 1). Trainees recognised the importance of non-technical skills and device training within the robotic operating environment. Both live operating and the history of robotics was deemed an unnecessary component of a PPCRC. Emergency scenarios and how to deal with them (72.1%) and suturing (67.4%) failed to achieved consensus but were deemed important by the steering committee in Phase 4. The use of ISCP procedure-based assessments achieved consensus in the first round (81%) supporting that any new curriculum should align with current reporting practices.

### Target groups and delivery

A standardised PPCRC would be advantageous to all trainees at or above the level of core surgical training (or equivalent) in the UK and Ireland and exposure should not be limited to only the senior years of the training programme (Table 4).

### Objective metrics, benchmarking and assessment

Participants supported that robotic cases should be deemed acceptable for index cases in higher surgical training programmes in the UK and Ireland (Table 5) and logbooks and workplace-based assessments should be updated to reflect this. Completion of core robotics skills training should be included in curriculae and required for completion of training and video assessments should be analysed with a validated, standardised scoring system.

## Recommendations

Based on the above findings, the authors make the following recommendations on the requirement and components of PPCRC:

1. A PPCRC integrating into surgical training in an inclusive and deliverable way would focus on the first three steps of training, not inclusive of procedural training and ensure all trainees receive standardised and validated training in the basics of robotic surgery
2. A universal PPCRC should focus on three main areas of training: e-learning, device training and simulation (virtual and dry lab).
3. A formal PPCRC should commence at the level of core surgical trainee or SHO or equivalent level of experience but exposure prior to this stage of trainings to be encouraged.
4. Assessment standards and ability to progress to procedural-based training should be clearly defined, standardised and validated. It should be collaborative to avoid duplication.
5. Training requirements in understanding the hardware technology as well as operative technique is required for all individual robotic surgery systems should be included in a PPCRS prior to live operating.
6. A PPCRC will benefit from a structured, proficiency-based curriculum that incorporates technical and non-technical skills training while interacting with the multi-disciplinary team.
7. A PPCRC should be aligned to ISCP and crowdsourced feedback should be considered.
8. Certain components of a PPCRC can be delivered at an individual level; however, robust collaborative local and national level training solutions are required.
9. Royal Colleges, educational authorities, industry and trainee and trainer stakeholders should all be consulted on the components of a PPCRC.

## Discussion

Using the Delphi methodology, we have achieved multispecialty consensus among trainees to develop and reach content validation for the requirements and components of a PPCRC in the UK and Ireland. There was 100% response from all three rounds and 97.7% agreement that a standardised PPCRC would be advantageous to training and that, independent of speciality, there should be a common approach (95.5% agreement).

Global adoption of robotic assisted surgery (RAS) in clinical practice is rapidly growing with clear evidence of this trend in the UK and Ireland [50, 51]. However, the current UK and Ireland Training Speciality curriculae contain no reference or training outcomes specific to RAST and trainee surgeons broadly remain robotic naïve until completion of certificate of training (CCT), where they are then expected to rapidly adopt procedure-based curricula that is significantly industry-driven and ad hoc delivered without universal access [31, 46, 52–56]. This is in direct contrast to training surgeons in the US [57].

The authors and Delphi participants believe that a PPCRC, integrated in parallel to current surgical training structures would improve surgical trainee literacy and skills in robotic surgery and could be delivered universally regardless of geographical location, surgical specialty and stage of training. This in turn would increase training opportunities in robotic surgery training for all surgical trainees and better prepare the future surgical workforce. As the adoption of robotic surgery is exponentially increasing, it is important that there is a mechanism in place to ensure the future consultant workforce have the foundational training required to be able to adapt to both robotics and digital surgery in real-time.

Consensus was reached in multiple areas: 1. Experience and Exposure, 2. Access and context, 3. Curriculum Components, 4 Target Groups and Delivery, 5. Objective Metrics, Benchmarking and Assessment. Trainees should have access to multiple industry e-learning curricula for common clinically integrated systems until a bespoke design of universal e-learning is achieved. Robotic technical skills training should be broken down into a PPCRC and procedural training (Fig. 1). By separating these two aspects we negate some of the limitations to access due to bottle necks in training related to lack of expertise in new technologies. This also fits with a proficiency-based progression approach as procedural training builds on the foundation knowledge and skills from a PPCRC [16]. Acquisition of robotic surgical skills by junior surgical trainees is often hindered by time pressure, financial imperatives and access to both robotic kit and patients undergoing robotic surgery. Robotic simulation training offers an attractive solution, because it allows trainees to learn in a safe, controlled, and standardised environment [58, 59]. Future procedural training will also likely incorporate VR simulation, so this approach also gives exposure to future novel training modalities [60].

Surgical training environments include classroom instruction, e-learning and practical training, in both the operating room and simulation. Surgical training focuses not just on technical and procedural instruction, but also training in non-technical skills, including crisis (emergency) management, decision making, leadership and communication, that benefits the team performance. In robotic surgery it is crucial to successfully complete device training and basic skills training prior to commencing advanced procedural training on patients. By utilising objective scores there are also opportunities for credentialing in robotic surgery that will help further guarantee patient safety through agreed processes of quality control. There is debate around whether this should be achievable (in part of in full) prior to CCT [61, 62].

A proficiency-based progression approach to training with objective metrics and benchmarks will give safety assurances to training. The curricula should be standardised and will require agreement from societies and education of trainers to achieve widespread adoption while delivering consistent content and assessments [16].

Kinematic data or automated performance metrics (APMs) (instrument and endoscopic camera motion tracking and events data, such as energy usage) can be recorded during robotic surgery and can collect large amounts of data, related to the surgeon’s performance. Machine learning, which is a form of artificial intelligence, relies on computer algorithms and large volumes of data to “learn” and recognize broad patterns that are often imperceptible to human reviewers. Machine learning (ML) algorithms have been used to process these large volumes of automatically collected data from robotic surgery and have been shown to correlate with patient outcomes [63]. Objective automated scores of surgical performance have huge potential to assess surgeons, predict patient outcomes and in the near future, give opportunities to personalize surgeon training. However, as the role of AI in healthcare continues to expand there is increasing awareness of the potential pitfalls of AI and the need for guidance to avoid them [64].

Crowdsourced feedback appears to be a cost effective and efficient way to assess surgical performance in procedural training, with results comparable to expert feedback, when assessing technical skills. However, inter-rater reliability is poor and further work is needed to increase consistency in evaluations, to explore sources of discrepant assessments between surgeons and crowds, and to identify optimal populations and novel applications for this technology [65].

It is recognised that robotic training curriculums often focus on technical skills training and lack training in the area of non-technical skills (NTS). For trainees to attain the requisite knowledge and skills to provide safe and effective surgical care, robotic surgical training will benefit from a structured, proficiency-based curriculum that incorporates technical and non-technical skills training. A growing awareness of the importance of cognitive and NTS will encourage further development of NTS training and inclusion in curricula [66].

## Limitations

It is important to acknowledge that the data presented in this manuscript is a consensus opinion and not based on previously published evidence. While surgical trainees are significant stakeholders in surgical training and as end users in surgical training are in a unique position to identify solutions and obstacles to practical introduction of robotic surgery training opportunities there are other significant stakeholders that were not involved in this consensus. All participants also understand the current training crisis that we are experiencing in our healthcare systems with 97% of participants reporting a significant disruption to their training. However, there is still an appetite and desire to continue to evolve and ensure that we do continue to grow and develop new and innovative surgical techniques including robotic surgery for the betterment of patient care.

## Conclusion

Robotic surgery practice is increasing, therefore, access to standardised and validated training should also increase. It is crucial that surgical trainees are prepared for this practice trend using a defined curriculum with a clear start and end point and appropriate assessment and benchmarking. Using the Delphi methodology, we achieved multispecialty consensus among trainees to develop and reach content validation for the requirements and components of a PPCRC in the UK and Ireland. This guidance will benefit from further validation following implementation.

## Supplementary Information

Below is the link to the electronic supplementary material.

Supplementary file1 (DOCX 41 KB)

## Data Availability

All data is available on request to the corresponding author.
