# Supplementary material for: Utilising an accelerated Delphi process to develop consensus on the requirement and components of a pre-procedural core robotic surgery curriculum
Source: J Robot Surg. 2023 Feb 9;17(4):1443–55. doi: 10.1007/s11701-022-01518-2 (PMC9909133; doi:10.1007/s11701-022-01518-2)
Supplement: Supplementary file 1 — Supplementary file1 (DOCX 41 KB) [file 11701_2022_1518_MOESM1_ESM.docx]

**Supplementary Table 1 – All Statements**

| **Item** | **Statement** | **Agree (%)** | **Disagree (%)** | **Achieved Consensus** | **Round Achieved** |
| --- | --- | --- | --- | --- | --- |
| **Experience & Exposure to Robotic Surgery** | | | | | |
| 1 | Robotic surgery can support an increase in delivery of minimally invasive surgery | 95.5 | 4.5 | YES | 1 |
| 2 | Robotic surgery can enable microsurgical techniques in surgery | 97.7 | 2.3 | YES | 1 |
| 3 | Robotic surgery can enable automated data collection on individual surgical performance | 93.0 | 7.0 | YES | 1 |
| 4 | Robotic surgery currently is relevant to your preferred speciality | 76.6 | 23.4 | NO | - |
| 5 | Robotic surgery will be relevant to your preferred speciality | 95.3 | 4.7 | YES | 1 |
| 6 | You foresee robotic surgery as part of your future consultant practice | 81.4 | 18.6 | YES | 2 |
|  | *Integration of robotic surgery training could have a negative impact on your overall surgical training due to* |  | | | |
| 7.1 | Consultants still on robotic learning curve resulting in less learning opportunities | 86.0 | 14.0 | YES | 2 |
| 7.2 | Compete with training opportunities in laparoscopic or open surgery | 72.1 | 27.9 | NO | - |
| 7.3 | Less complex cases selected to be performed robotically that would have offered a good training opportunity in open or laparoscopic | 46.5 | 53.5 | NO | - |
| 7.4 | Longer duration of cases result in reduced training case volume | 39.5 | 60.5 | NO | - |
| 7.5 | Compete with more valuable learning opportunities in current training environment | 18.6 | 81.4 | YES | 1 |
| **Item** | **Statement** | **Agree (%)** | **Disagree (%)** | **Achieved Consensus** | **Round Achieved** |
| **Access and Context** | | | | | |
| 8 | Where robotic surgery training is not available in all hospitals, a deanery/regional based structure should be established for delivering training in robotic surgery to increase access | 95.3 | 4.7 | YES | 1 |
| 9 | Where robotic surgery training is available in hospitals, a deanery/regional based structure should be established for delivering training in robotic surgery and further increase access | 83.7 | 16.3 | YES | 1 |
| 10 | Monthly access to a regional hub for core robotic surgery training is reasonable | 86.0 | 14.0 | YES | 2 |
| 11 | Regional hubs should be supervised by robotic surgery trainers if they were an option | 93.0 | 7.0 | YES | 1 |
| 12 | Individual robotic surgery trainers should be accredited | 93.0 | 7.0 | YES | 1 |
| 13 | Training centres and training hospitals should be assessed and accredited via a recognised education entity, like a royal college or society | 90.7 | 9.3 | YES | 1 |
|  | Deanery/regional base approach to robotic surgery training should comprise |  | | | |
| 14.1 | *Device training in regional hub* | 86.0 | 14.0 | YES | 1 |
| 14.2 | *Non-technical skills and operative training in regional hub* | 74.4 | 25.6 | NO | - |
| 14.3 | *Skills training in regional hub* | 72.1 | 27.9 | NO | - |
| 14.4 | *E-learning (any site)* | 62.8 | 37.2 | NO | - |
| 14.5 | *Non-technical skills and operative training any site* | 51.2 | 48.8 | NO | - |
|  | During which times should trainees attend regional hubs if they were available through |  | | | |
| 15.1 | *Time-tables as part of working day* | 88.4 | 11.6 | YES | 2 |
| 15.2 | *Approved educational leave* | 81.4 | 18.6 | YES | 3 |
| 15.3 | *Zero days* | 4.7 | 95.3 | YES | 1 |
| 15.4 | *Free time* | 4.7 | 95.3 | YES | 1 |
|  | If regional hubs existed, what duration of access would be preferable |  | | | |
| 16.1 | *Full day* | 76.4 | 23.6 | NO | - |
| 16.2 | *Half day* | 34.9 | 65.1 | NO | - |
|  | A reasonable distance to travel to a regional hub is |  | | | |
| 17.1 | *<20km* | 27.9 | 72.1 | - | - |
| 17.2 | *<50km* | 83.7 | 16.3 | YES | 3 |
| 17.3 | *<100km* | 2.3 | - | - | - |
| **Item** | **Statement** | **Agree (%)** | **Disagree (%)** | **Achieved Consensus** | **Round Achieved** |
| **Curriculum Components** | | | | | |
|  | Components of a core robotic surgery curriculum should include |  | | | |
| 18.1 | *Virtual simulation training and e-learning* | 90.7 | 9.3 | YES | 1 |
| 18.2 | *Baseline evaluation* | 83.7 | 16.3 | YES | 2 |
| 18.3 | *Device training* | 88.4 | 11.6 | YES | 1 |
| 18.4 | *Dry lab training* | 86.0 | 14.0 | YES | 1 |
| 18.5 | *e-learning* | 69.8 | 30.2 | NO | - |
| 18.6 | *First assistance training* | 69.8 | 30.2 | NO | - |
| 18.7 | *Wet lab training* | 51.2 | 48.8 | NO | - |
| 18.8 | *History of robotics* | 18.6 | 81.4 | YES | 1 |
| 18.9 | *Live operating* | 16.3 | 83.7 | YES | 1 |
| 19 | Division of robotic surgery training into different progressive phases to include: e-learning, device training, basic skills and procedural training is an effective approach | 95.3 | 4.7 | YES | 1 |
| 20 | A core robotic surgery curriculum should include: e-learning, device training and basic skills | 97.7 | 2.3 | YES | 1 |
| 21 | Following successful completion of core robotic surgery training, a trainee should be able to proceed to procedural training in their preferred specialty | 95.3 | 4.7 | YES | 1 |
| 22 | All training should include objective metrics to assess progression to a defined level | 97.7 | 2.3 | YES | 1 |
| 23 | Core robotic surgery training should be linked to proficiency based progression, with 'bench marking' of pass/fail levels? | 86.0 | 14.0 | YES | 2 |
| 24 | Trainees benefit from validated objective scoring systems to provide consistent feedback | 93.0 | 7.0 | YES | 1 |
| 25 | Benchmarking of an acceptable standard of performance for trainees should be defined within the curriculum | 88.4 | 11.6 | YES | 1 |
| 26 | Trainees should pass core robotic training before commencing advanced procedural training | 95.3 | 4.7 | YES | 1 |
| 27 | All training should include baseline evaluation for assessment of training needs | 93.0 | 7.0 | YES |  |
| 28 | Baseline evaluation could enable different entrance levels to the core training, that takes into account the individuals current exposure to robotic surgery training | 86.0 | 14.0 | YES | 1 |
| 29 | For novice trainees, online e-learning and completion of a baseline evaluation should be a pre-requisite proceeding to 'practical' core robotic training in the form of device training and basic skills | 81.4 | 18.6 | YES | 1 |
|  | Baseline evaluation should include |  | | | |
| 30.1 | *VR Simulation modules* | 83.7 | 16.3 | YES | 1 |
| 30.2 | *Dry lab training* | 67.4 | 32.6 | NO | - |
| 30.3 | *Written test following e-learning* | 53.5 | 46.5 | NO | - |
| 30.4 | *First assistant* | 41.9 | 58.1 | NO | - |
| 30.5 | *Wet lab training* | 32.6 | 67.4 | NO | - |
| 30.6 | *OR team training* | 20.9 | 79.1 | NO | - |
|  | E-learning should include |  | | | |
| 31.1 | *Description of hardware in various systems* | 90.7 | 9.3 | YES | 1 |
| 31.2 | *Info on patient selection and preparation* | 86.0 | 14.0 | YES | 1 |
| 31.3 | *Info on trouble shooting* | 90.7 | 9.3 | YES | 1 |
| 31.4 | *How to dock* | 93.0 | 7.0 | YES | 1 |
| 31.5 | *Emergency scenarios and how to deal with them* | 72.1 | 27.9 | NO | - |
| 31.6 | *Non-technical skills* | 53.5 | 46.5 | NO | - |
| 31.7 | *Operative team training* | 46.5 | 53.5 | NO | - |
| 31.8 | *History and development of robotic surgery* | 18.6 | 81.4 | YES | 1 |
|  | Technical core robotic skills training should include |  | | | |
| 32.1 | *Two-handed movements* | 97.7 | 2.3 | YES | 1 |
| 32.2 | *Camera directioning* | 97.7 | 2.3 | YES | 1 |
| 32.3 | *Basic movements* | 95.3 | 4.7 | YES | 1 |
| 32.4 | *Tissue dissection* | 81.4 | 18.6 | YES | 1 |
| 32.5 | *Knot tying* | 83.7 | 16.3 | YES | 1 |
| 32.6 | *Suturing (67.4%)* | 67.4 | 32.6 | NO | - |
| 32.7 | *Safe use of diathermy* | 67.4 | 32.6 | NO | - |
| 32.8 | *Retraction using 4^th^ arm* | 58.1 | 41.9 | NO | - |
| 33 | Formal device training should be mandatory in a core robotic surgery curriculum | 90.7 | 9.3 | YES | 1 |
| 34 | Training on each type of robotic surgery device is required before operating on a patient | 93.0 | 7.0 | YES | 1 |
| 35 | Non-technical skills training should be a component of a core robotic surgery curriculum | 88.4 | 11.6 | YES | 1 |
|  | Non-technical skills should include |  | | | |
| 36.1 | *Situation awareness training* | 90.7 | 9.3 | YES | 1 |
| 36.2 | *Operative team training* | 86.0 | 14.0 | YES | 2 |
| 36.3 | *Communication* | 88.4 | 11.6 | YES | 2 |
| 36.4 | *Emergency scenarios* | 83.7 | 16.3 | YES | 1 |
| 36.5 | *Decision making* | 58.1 | 41.9 | NO | - |
| 36.6 | *Leadership* | 44.2 | 55.8 | NO | - |
| 36.7 | *Video performance with feedback* | 34.9 | 65.1 | NO | - |
| 36.8 | *Cognitive skills training* | 27.9 | 72.1 | NO | - |
| 37 | Operative team training should be a component of a core robotic surgery curriculum | 90.7 | 9.3 | YES | 1 |
|  | Operative team training should include |  | | | |
| 38.1 | *Docking* | 92.9 | 7.1 | YES | 1 |
| 38.2 | *Emergency scenarios* | 90.5 | 9.5 | YES | 1 |
| 38.3 | *Bedside assistance* | 85.7 | 14.3 | YES | 1 |
| 38.4 | *Team decision making* | 78.6 | 21.4 | NO | - |
| 38.5 | *Theatre team efficiency training* | 55.8 | 44.2 | NO | - |
| 38.6 | *Patient turnaround* | 39.5 | 60.5 | NO | - |
| 39 | Non-technical skills training and team training should be evaluated with a scoring system? | 83.7 | 16.3 | YES | 3 |
| 40 | Non-technical skills and team training can be sufficiently assessed with NOTSS | 88.4 | 11.6 | YES | 1 |
| **Item** | **Statement** | **Agree (%)** | **Disagree (%)** | **Achieved Consensus** | **Round Achieved** |
| **Target Groups & Delivery** | | | | | |
| 41 | A standardised core robotic training curriculum will be advantageous to training | 97.7 | 2.3 | YES | 1 |
| 42 | Independent of specialty,there should be a common approach for core robotic surgery training | 95.3 | 4.7 | YES | 1 |
| 43 | A core robotic surgery training programme should bring you to point of procedural training | 88.4 | 11.6 | YES | 1 |
|  | A core robotic curriculum should be available to the following |  | | | |
| 44.1 | *Core surgical trainees (or equivalent)* | 88.4 | 11.6 | YES | 1 |
| 44.2 | *Registrars (or equivalent)* | 97.7 | 2.3 | YES | 1 |
| 44.3 | *Fellows (or equivalent)* | 83.7 | 16.3 | YES | 1 |
| 44.4 | *Robot naïve surgeons* | 83.7 | 16.3 | YES | 1 |
| 44.5 | *Laparoscopic surgeons* | 81.4 | 18.6 | YES | 1 |
| 44.6 | *Open Surgeons* | 69.8 | 30.2 | NO | - |
| 44.7 | *Theatre staff* | 32.6 | 67.4 | NO | - |
| 44.8 | *Extended surgical team* | 23.3 | 76.7 | NO | - |
| 44.9 | *Foundation year doctors* | 16.3 | 83.7 | YES | 1 |
| 44.10 | *Medical student* | 20.9 | 79.1 | NO | - |
|  | A core robotic curriculum should consider the experience of the following |  | | | |
| 45.1 | *Core surgical trainees (or equivalent)* | 90.7 | 9.3 | YES | 1 |
| 45.2 | *Registrars (or equivalent)* | 95.3 | 4.7 | YES | 1 |
| 45.3 | *Fellows (or equivalent)* | 81.4 | 18.6 | YES | 1 |
| 45.4 | *Robot naïve surgeons* | 91.4 | 8.6 | YES | 1 |
| 45.5 | *Laparoscopic surgeons* | 86.0 | 14.0 | YES | 1 |
| 45.6 | *Open Surgeons* | 83.7 | 16.3 | YES | 1 |
| 45.7 | *Theatre staff* | 27.9 | 72.1 | NO | - |
| 45.8 | *Extended surgical team* | 18.6 | 81.4 | YES | 1 |
| 45.9 | *Foundation year doctors* | 14.0 | 86.0 | YES | 1 |
| 45.10 | *Medical student* | 7.0 | 93.0 | YES | 1 |
| 46 | Does robotic surgery only have a role in senior years of training? (Answer - NO) | 90.7 | 9.3 | YES | 2 |
| 47 | Robotic surgery training should only be delivered post-CCT (Answer - DISAGREE) | 93.0 | 7.0 | YES | 1 |
| 48 | Training in robotic surgery is beneficial in earlier years of surgical training (Answer - AGREE) | 90.7 | 9.3 | YES | 1 |
|  | Training in robotic surgery is beneficial in early years of training due to |  | | | |
| 50.1 | *Familiarity with basic principles in robotics* | 83.7 | 16.3 | YES | 1 |
| 50.2 | *Development of robotic surgery non-technical skills* | 79.1 | 20.9 | NO | - |
| 50.3 | *Development of laparoscopic skills as first assistance* | 72.1 | 27.9 | NO | - |
| 50.4 | *Experience of implementation of innovation in healthcare* | 30.2 | 69.8 | NO | - |
| 50.5 | *Bedside operative case management* | 20.9 | 79.1 | NO | - |
| **Item** | **Statement** | **Agree (%)** | **Disagree (%)** | **Achieved Consensus** | **Round Achieved** |
| **Objective Metrics, Benchmarking & Assessment** | | | | | |
| 51 | Cases performed robotically should be accepted for indicative numbers for index cases in surgical training | 83.7 | 16.3 | YES | 1 |
| 52 | Should standard logbook programmes facilitate an option for recording robotic approach to standard surgical procedures | 88.1 | 11.9 | YES | 1 |
| 53 | Surgical curricula should reference the role of robotic surgery and guide on its place in training | 90.7 | 9.3 | YES | 1 |
| 54 | Current curricula approved Procedure Based Assessments (PBAs) should be adjusted (where appropriate) to be suitable to assess robotic approach to index cases | 90.7 | 9.3 | YES | 1 |
| 55 | Specialty-relevant index procedure PBAs should be completed in simulation prior to live-operating? | 81.4 | 18.6 | YES | 2 |
| 56 | Completion of core robotic surgery skills training should be considered as an approved surgical training course for certification | 83.7 | 16.3 | YES | 1 |
| 57 | Trainees should receive a 'sign-off' following completion of core robotic surgery skills | 95.3 | 4.7 | YES | 1 |
|  | Final assessment for ‘sign-off’ should include |  | | | |
| 58.1 | *VR simulation modules* | 97.7 | 2.3 | YES | 2 |
| 58,2 | *Dry lab skills* | 79.1 | 20.9 | NO | - |
| 58.3 | *Written, knowledge based, test following e-learning* | 53.5 | 46.5 | NO | - |
| 58.4 | *Wet lab skills* | 44.2 | 55.8 | NO | - |
| 58.5 | *First assistant evaluation* | 32.2 | 67.8 | NO | - |
| 58.6 | *Non-technical skills* | 30.2 | 69.8 | NO | - |
| 59 | *Core robotic surgery skills training should be formally built into surgical curriculum?* | 86.0 | 14.0 | YES | 2 |
| 60 | *Robotic surgery training could have any negative impact on your overall surgical training* | 83.7 | 16.3 | YES | 3 |
|  | Technical core robotic skills training assessments should include |  | | | |
| 61.1 | *ISCP OBAs* | 81.0 | 19.0 | YES | 1 |
| 61.2 | *Objective score with defined task steps and errors to avoid* | 46.5 | 53.5 | NO | - |
| 61.3 | *Video performance analysis with feedback* | 34.9 | 65.1 | NO | - |
| 61.4 | *Machine-based metrics* | 23.3 | 76.7 | NO | - |
| 61.5 | *GEARS* | 7.0 | 93.0 | YES | 1 |
| 62 | *Videos should be analysed with a validated standardised objective scoring system* | 90.7 | 9.3 | YES | 1 |
|  | Scoring systems for video analysis should include |  | | | |
| 63.1 | *Combination of subjective and objective scoring systems* | 79.1 | 20.9 | NO | - |
| 63.2 | *GEARS* | 41.9 | 58.1 | NO | - |
| 63.3 | *Objective scoring system that measures visual cues, technical errors and technical events* | 23.3 | 76.7 | NO | - |
| 63.4 | *OSATS* | 20.9 | 79.1 | NO | - |
| 63.5 | *ABS operative performance rating* | 2.3 | 97.7 | YES | 1 |
| 63.6 | *At least two 'experts' should analyse and review video performance* | 81.4 | 18.6 | YES | 1 |
